# 

**Published:** 1891-01-10

**Authors:** 


### NOTICE TO CORRESPONDENTS.

All MS., Letters, Books for Review, and other matters intended for the Editor should be addressed The Editor, The Lodge, Porchester Square, London, W.

The Editor cannot undertake to return rejected M.S., even when accompanied by stamped directed envelope.

### Notice to Advertisers and Subscribers.

All Advertisements, Orders for Copies of The Hospital, and Business Communications should be addressed to The Publisher, *not to the Editor*, The Hospital, 140, Strand, W.C.

The Cost of Subscription, post free, is as follows:—Per week, 1½d.; per quarter, 1s. 8d.; per half-year, 3s. 3d.; per annum, 6s. 6d. Foreign:—Per annum, 8s. 8d.; payable, in all cases, in advance.

### Bound Volumes of "The Hospital."

Vol. VI., containing full page portraits of T.R.H. the Prince and Princess of Wales, and Miss Florence Nightingale, is still obtainable.

Vol. VII. and all the earlier volumes are also for sale.

Orders will be received for Vol. VIII., 4s. post free. Cases for binding may be had of the Publisher, at 1s. 6d. each.

### To Residents, Secretaries, and Matrons.

The Editor will be glad to have the Regulations and each Report as issued by Asylums, Hospitals, Convalescent and Nursing Institutions, as well as those of other Charities, and an early copy in duplicate of all Appeals and Festival Notices, etc., addressed to *The Editor, The Lodge, Porchester Square, London, W.* Any superintendent, secretary, matron, or other chief official who regularly sends such information may be registered, on application to the Publisher, to receive free of cost a cover for binding The Hospital in half-yearly volumes.

### BURDETT'S HOSPITAL ANNUAL FOR 1890

May be obtained of the Publisher, The Hospital Offices, 140, Strand, W.C., price 2s. 6d. limp boards, or 3s. 6d. cloth. All orders must be accompanied by a remittance.

### SCRAPS AND GLEANINGS.

Ten thousand dinners were distributed to the poor on Christmas Eve in Harley Street.

The potters at Doulton's manufactory are still out on strike. The works may shortly be closed.

Viscount Portman and Sir Edward Guinness have each given £100 to the Irish Distressed Ladies' Fund.

Mr. Joseph Duck, whose birth register was recently discovered in London, had died at the age of 102 years.

A daring robbery has been committed at Guy's Hospital. Seven watches, besides other jewellery, were taken.

Dr. Robinson, of Maine, has examined 100 wall papers, and found a dangerous amount of arsenic in three of them.

The influenza has broken out again at Vienna. The epidemic is, however, much less severe than that of last winter.

An English medical man, the proprietor of a sanatorium at Nice, proposes to establish a grape cure sanatorium at Mildura, Victoria.

The outbreak of diphtheria at Surbiton, which has caused three or four deaths, has been traced, like that at Croydon, to the milk supply.

The Lord Mayor's fund for the relief of the widows and orphans of the crew of the "Serpent" amounted, on Wednesday, to about £2,000.

The sum of £250, being part of the receipts of the Norfolk and Norwich Musical Festival, has been divided among the hospitals and convalescent homes of Norfolk.

A horse has died from hydrophobia, the result of injuries inflicted by a mad dog. A child who was bitten by the same dog is being treated by M. Pasteur, and is doing well.

Another fatal electric light accident occurred at Denver, U.S.A. A line man, engaged in repairing a wire at the top of a pole, touched a live wire, and was killed on the spot.

An epidemic of small-pox has broken out at Haderslebon, where several persons have died of the disease, which is spreading in an alarming manner; chiefly among the poor.

The Marquis of Salisbury has become vice-president of the National Health Society, and has contributed £10 10s. to the funds. The society does much sanitary work among the poor.

At a recent meeting of the Paris Academy of Medicine, M. Motais, of Angers, maintained that myopy, or shortness of sight, was a defect incident to civilisation, or an artificial condition of life.

During the past two months Mr. Patrick Donahoe has sent nearly £10,000 to Ireland on behalf of Irish servant girls in America. The amount exceeds the record of any previous Christmas time.

The doctor at the Madrid Hospital reports that the condition of the two persons suffering from leprosy, who, at their own request, were inoculated with Dr. Koch's lymph, could not be more satisfactory.

A woman has died at Yeovil at the age of 108. She had been bedridden for many years, and was both blind and deaf, but she retained her mental faculties to the end. Her daughter, who attended to her, is 80 years of age.

Dr. Illingworth has designed a medical practitioner's cash book, which he has registered. It is published by Thomas Broadley, Gutenberg Works, near Accrington, price 27s., half-bound Russian morocco, and lettered.

The Skinners' Company have sent ten guineas, and the Joiners' Company five guineas, to the Bethnal Green Free Library, making the fifth grant made by each of these companies to this institution, which is supported by voluntary effort.

The Board of Agriculture have decided to withdraw the Dog Muzzling order. On and after January 1st no dog equipped with a collar bearing the name and address of the owner will need to be muzzled within the metropolitan area.

It has been proposed to call Professor Koch's lymph "Kochine," but this is objected to by all scientific authorities, because the termination "ine" designates the alkaline basis of vegetables, as in the words quinine, strychnine, and so forth.

Reports having appeared in the English newspapers to the effect that cholera exists in Palestine, Messrs. Cook state that Palestine is entirely free from cholera, and that there is no quarantine on ships calling at Jaffa, Caija, or Beyrout.

A requisition is being got up to the Mayor of Birmingham, asking him to call a public meeting similar to the one recently held in the Guildhall, London, to protest against the inhuman treatment of the five million Jews in the Russian Empire.

Late on Wednesday afternoon the London and India Dock Joint Committee issued a circular, warning their employés that all who had been employed by the committee less than ten years would not be required after December 31st, and advising them to seek other work. The notice affects some hundreds of men.

At the fortnightly meeting of the Altrincham Rural Sanitary Authority, Dr. Fox, medical officer, reported an outbreak of scarlet fever at Northenden, which had become epidemic in character. Every precaution had been taken to prevent its spread, and he should investigate and report fully at the next meeting.

It is officially announced at Berlin that the Emperor has assumed the patronage of the Royal Sailors' Home at Portsmouth, two wards of which have consequently received the names of "Emperor William II." and "Hohenzollern" respectively. In future petty officers and men of the German Navy are to be admitted into the Home at any time, and on the same conditions as British seamen.

Mr. Lennox Browne gave a clinical lecture at the Central London Throat, Nose, and Ear Hospital on "Koch's Remedy in Tuberculosis of the Throat." While declaring himself an enthusiastic disciple of Koch, he deprecated the least departure from the rigid details of procedure enjoined by the discoverer, or the exaggeration of the really modest hopes of benefit which he had promised.

### THE STUDENT OF MEDICINE.

[All communications for this Supplement should be addressed to THE EDITOR of THE HOSPITAL, at 140, Strand, with the words, "Students' Column," written in the left hand top corner of the envelope.]

### STUDENTS' MEDICAL SOCIETIES.

### Guy's Hospital Pupil's Physical Society.

A meeting was held on Saturday, December 13th, when Mr. A. S. Wohlmann read a paper on "Recent Poisoning Cases in Guy's." He reviewed and commented on the poisoning cases, sixty-two in number, which had been taken into John and Miriam wards during 1887-89. Only seven terminated fatally, of which four died from the effects of soldering fluid, two from opium, and one from oxalic acid. Messrs. F. G. Hopkins, Guy Mackeson, A. M. Daldy, A. T. Rake, and T. M. Gill joined in the discussion which followed, and the meeting was closed by a hearty vote of thanks to Mr. Wohlmann for his excellent paper.

### King's College Medical Society.

INAUGURAL ADDRESS (*continued*).

"A Short Description of a New Modification of the Operation of Inquinal or Iliac Colotomy."

Professor Rose, after the preliminary remarks reported in our issue of December 27th, passed on to the second portion of his address, and proceeded as follows: The establishment of an artificial anus by left lumbar colotomy (or Amussat's operation) was in considerable favour till within the last ten years. But as the dread of opening the peritoneum has passed away with the increased confidence in antiseptics, so the anterior operation has grown in favour, and the peritoneum has been shown by its properties of absorption and of rapidly forming adhesions to be an advantage, and therefore forms a great *prima facie* argument in favour of the anterior operation. There are, however, additional advantages in the anterior operation, among the more important ones are the following—(1) The position of the artificial opening is more under the patient's control, and more suitable for the adaptation of a retention apparatus. If the opening be in the loin it is not an easy matter for the patient to keep himself clean. (2). There is a greater facility in operating through the anterior-abdominal wall; there is here plenty of room, and a clean opening is made through the abdominal parietes without any fear of bruising or damaging the subserous cellular tissue. (3). The depth of the lumbar wound as compared with that of the iliac operation increases both the difficulty and the danger of the former. The colon cannot be defined with so much certainty, and the traction upon the stitches of fixation is greater in the lumbar operation. (4). Then again there is always the possibility of a well-marked meso-colon existing which would involve an opening into the peritoneal cavity under less advantageous circumstances, and might possibly lead to a different portion of the intestine being opened to that desired. (5). For purposes of diagnosis and prognosis the inguinal operation undoubtedly offers this great advantage, altogether unpossessed by the lumbar operation; that the hand can be introduced through the opening and a thorough exploration of the abdominal contents accomplished. As a proof of this Professor Rose mentioned that in two recent cases at the hospital he was enabled to diagnose the probable malignant nature of the cause of the obstruction and its exact situation. (6). Again, one of the great after troubles of colotomy consists in the retraction of the gut, rendering the action of the artificial anus imperfect, by allowing the passage of fæcal matter into the distal opening, and it was owing to this circumstance that Professor Rose was induced to modify the operation of inguinal colotomy in a manner which he detailed later on. On the other hand, lumbar colotomy has its advantages. (1). When the demand for opening the intestine is urgent and immediate by lumbar colotomy the risk of intra peritoneal fæcal extravasation is avoided. (2.) There is in it less tendency to relapse subsequently, whereas the ordinary inguinal or iliac operation is unquestionably open to this serious objection; but Professor Rose stated that this objection can be entirely prevented by the modification which he has introduced, and which he then described as follows: With all antiseptic precautions an incision should be made transversely to a line drawn from the umbilicus to the anterior superior iliac spine of the left side, a little nearer the anterior iliac spine than a point midway between the two landmarks; the abdominal wall should be carefully dissected through, including the parietal peritoneum. The parietal peritoneum should now be stitched to the skin, and the stitches must not include any of the intermediate layers between those tissues. The descending colon is next sought for, and the whole circumference of it pulled out of the wound, so that a complete coil of the intestine lies outside the abdominal cavity; this must then be fixed in this position by a double, deep, strong catgut suture, which is passed, first of all, through the abdominal parietes on one side of the wound, then through the meso-colon, this being underneath the extended gut, and then through the parietes of the other side of the wound. The same catgut stitch is then repassed in the same manner through the meso-colon and abdominal parietes, about half an inch distant from the initial passage, and brought out half an inch distant from its starting point; the two ends of it being on the same side are then tightened and ligatured, the tightening bringing the parietal peritoneum of both sides of the wound into close contact, thus preventing completely any tendency of the extended bowel to retract. The colon may then be sewn to the peritoneal cutaneous margin with fine catgut stitches. The wound is then antiseptically dressed, and left, if possible, without opening the gut for five or six days. The advantages claimed for the operation were these: (1) Its simplicity. Here but one suture is passed and repassed through the abdominal parietes, the only point to be careful of is the avoidance of puncturing in the passage of the needle of a vessel in the meso-colon. (2) The formation of an efficient spur, and the complete separation of the upper from the lower opening. Professor Rose thus specially maintained that the distal opening should not be entirely excluded as in Madelung's operation, as the lower end of the bowel could not then be cleansed. There is in this method a diminished tendency to subsequent prolapse. Professor Rose then terminated his address by briefly referring to five cases in which he had performed this operation, in two of which it was deemed necessary to open the gut at the time of the operation, no bad result occurred which indicated any fæcal intra peritoneal extravasation. In one case on the third day the patient was seized with acute pain in the region of the wound and vomiting. The dressings were removed and a knuckle of small intestine was found to have protruded between the angle of the wound and the coil of large intestine drawn out at the operation; the knuckle of small intestine was carefully replaced and the patient was immediately relieved and made a very satisfactory recovery. Dr. Duffin proposed a vote of thanks to Professor Rose, and Professor Henry Smith seconded the proposal, in which he was supported by Messrs. Whitfield and E. L. Pritchard, the vote being carried unanimously amidst cheers.

### THE STUDENTS' BOOKSHELF.

In our review recently of the "Manual of Urine Testing" by Mr. John Scott, we omitted to mention that the book was published by W. Mullan and Sons, Belfast.

### PASS LISTS.

### Society of Apothecaries of London.

The following, having passed the qualifying Examination in Medicine, Surgery, and Midwifery, were granted the diploma of the society, entitling them to register and to practise in the same: C. E. Baxter, J. M. Bennett, E. Boardman, F. H. B. Brown, R. B. Eccles, W. A. Eden, A. A. Grosvenor, C. P. Lovell, J. Lupton, C. A. Morgan, B. Nauth, B. S. Norris, W. E. Passmore, F. C. Rogers, J. T. West, and C. S. Woodd.

In the Examination in Arts last month there were 190 candidates, of whom 16 received certificates, having passed in all the subjects required for registration as medical student. 109 passed in one or more of the subjects.

Surgery.—C. A. Beck, Bonn University and Middlesex Hospital; J. M. Bennett, Liverpool University College and Sheffield; E. Boardman, Madras University and Royal Free Hospital; F. H. B. Brown, B.A.Oxon. Oxford, and Guy's Hospital; J. H. Dempster, King's College; R. B. Eccles and A. Graydon, St. Thomas's Hospital: A. A. Grosvenor, B.A.Cantab., Cambridge and Guy's Hospital: S. Melville, University College, Liverpool; W. L. G. Morgan, St. Thomas's Hospital; J. S. Newington, Edinburgh University; B. S. Norris, Queen's College, Birmingham; W. E. Passmore. Westminster Hospital; H. de V. Stacpool, St. Mary's Hospital; J. T. West, Queen's College, Birmingham; C. S. Woodd, St. Bartholomew's Hospital.

Medicine, Forensic Medicine, and Midwifery.—C. E. Baxter, Sheffield and Charing Cross Hospital; F. H. B. Brown, B.A. Oxon., Oxford and Guy's Hospital; W. A. Eden, King's College; M. J. Houghton, Queen's College, Birmingham; C. P. Lovell, B.A. Oxon., Oxford and St. Thomas's Hospital; J. Lupton, Owens College, Manchester; C A Morgan, St. Thomas's Hospital; B. Nauth, L. M. S. Punjaub University, Lahore: B. S. Norris, Queen's College, Birmingham: L. E. Parkhurst, B.A.Oxon., Oxford and St. Mary's Hospital; F. C. Rogers, Owens College, Manchester; P. J. Ryan, University College; J. T. West, Owens College, Birmingham; C. S. Woodd, St. Bartholomew's Hospital.

The following passed in the subjects as indicated:—

Forensic Medicine and Midwifery.—M. H. Knapp, St. Mary's, Hospital.

Medicine.—C. A. Lapthorn, Middlesex Hospital; S. Melville, University College, Liverpool; C. T. Standring, King's College.

FORENSIC MEDICINE.—H. B. T. Symons, Aberdeen University and Charing Cross Hospital; A. L. Wykham, M.D., Havard University Washington.

### Faculty of Physicians and Surgeons at Glasgow.

At the December sittings of the Board of Examiners in Sanitary Science, the following candidates, having passed the necessary examinations, were admitted diplomates in Public Health: David P. Gage, L.R.C.P. and S. Ed., &c., Kilwinning; Hamilton C. Reid, M.B., C.M., Coatbridge; William Spence, M.B., C.M. Dollar.

### Society of Apothecaries.

The results of the Examination in Arts qualifying for registration as medical student, held in the hall of the society on the 5th and 6th ult., have just been published. There were 189 candidates, and from the Pass List it appears that 16 were placed in the second class, and 110 were certified as having passed in some subjects, but not in all. The next examination will be held on March 6th and 7th.

### ATHLETICS.

A PURELY AMATEUR TEAM TO VISIT THE UNITED STATES NEXT SEASON.—At the dinner of the London Athletic Club, held on Friday evening, Mr. W. B. Kent Hughes, the captain of the United Hospitals Athletic Club, in replying to the toast of "The Universities and Visitors," said that he considered it a disgrace for a team of so-called amateurs to visit America and contend at mere exhibition meetings whilst the amateur championships were being decided, and that it was the fault of the Amateur Athletic Association that better men were not tried. Next year Mr. Hughes contemplates visiting America with a team of Medicals to compete against Yale and Harvard Universities. At present the arrangements are not complete, but all those who have up to date been asked to join in the trip have gladly consented. As this tour will be conducted on purely amateur lines, the members of the team will, no doubt, receive a cordial reception from American amateur athletes.

AN OLD RECORD.—At a recent dinner the chairman, a well-known 'Varsity athlete, dilated upon swimming. In the course of an important speech, he remarked that Leander, who, most ordinary beings have read, swam across the Hellespont in search of his lady love, accomplished the distance in one hour and ten minutes. If this had been a recent performance a few fifths would have been tacked on, as well as the name of the watchmaker. Specialists are now hunting about for the name of the time-taker who noted Leander's record. His performance does not appear in the record books of the Amateur Swimming Association.

SWIMMING.—Owing to the thick coating of ice covering the Serpentine the annual swimming match which usually comes off on Christmas morning had to be put off. It was stated that a fairly good entry had been received.

### Football. ASSOCIATON RULES, DEC. 19.

The Cambridge University team continued their tour; when, at Preston, they lost to the North End club by eight goals to none. The turf was covered with snow.

### RUGBY RULES, DEC. 29TH.

*Edinburgh University v. Bikenhead Park.*—This match was played at Birkenhead in very wintry weather. P. H. Lockwood commenced play, and for some time the home team played a strictly defensive game. The University skipper, Pender, rendered a good account of himself, and Thom was within an hair's breadth of scoring. The visitors continued to press their opponents, but Birkenhead was so strong that but little headway could be made. On numerous occasions grand dribbles were made by J. Strang. Up to half time University had scored four minors. After changing ends Pender was repeatedly applauded for grand play, but he did not manage to score, although he made several magnificent runs. The way Strang led his forwards was a treat to witness, and he played a champion game. Birkenhead managed to force two minor points. Edinburgh University was victorious by four minors to two.

NORTH v. SOUTH.—This match was played at Leeds in the presence of about 9 000 visitors, and proved to be a most interesting game. The victory of the North over the South was not altogether unexpected, there were many changes in the fifteens originally chosen to play on the 20th, at Blackheath, the severe weather necessitating a removal of the fixture to Headingly, a new ground near Leeds. The afternoon was bright, and the ground was in good condition, having been protected from the frost by hay and straw. At half-past two, the South having lost the toss, kicked off, Rogers starting the ball. The North assumed the aggressive, and, after Valentine had nearly got in, Teothill scored the first try for the home team, and Coop placed a goal. The visitors carried several scrummages, while Christopherson and Morrison took the ball close up to the line, the latter seemed certain of scoring when Bromet promptly touched down. Twice the North compelled the South to touch down, while a further try was gained by Bromet. The Northern forwards were well supported by good back play, Berry and Cross taking a prominent part. Several fine runs were made by Lockwood and Valentine, but they were met with safe tackling by Johnson and the three-quarters. Leake, Morrison, and Christopherson carried the play into the home territory, and the last-named passed to Rotherham, who gained a try, which Woods converted into a goal for the South. Thus the North was only one point ahead of their opponents. The visitors tried hard to equalise matters, and the North to score further. Another try by Toothill gave the home team the advantage at half-time of one goal and two tries to one goal. Soon after changing ends the South touched down, a claim of a try by Bromet being overruled. The South for a time had rather the better of the play, in which the three-quarters were particularly prominent. Soon, however, the North again scored, another try being added by Lockwood. No other point was gained, and thus the North was victorious by one goal and three tries to one goal.

THE HOSPITAL CUP.—At a meeting of the United Hospitals Football Club the Cup Ties were drawn as follows: January 26th, St. Thomas's v. St. Mary's; January 27th, St. Bartholomew's v. Middlesex; January 29th, St. George's v. University; January 30th, Charing Cross v. Westminster; February 2nd, Guy's v. London.

### EVENTS FOR THE MONTH OF JANUARY.

[Items intended for this column must reach the office, 140, Strand W.C., not later than first post on Monday mornings.]

### Medical Societies.

ST. BARTHOLOMEW'S HOSPITAL ABERNETHIAN SOCIETY.—January 15th, Mr. Launcelot Andrews, M.B., on "Lead Poisoning." January 22nd, Mr. W. J. Walsham, F.R.C.S., on "Nasal Stenosis; its causation, symptoms, effects, and treatment." January 29th, Mr. E. J. Moore on the "Treatment of Plural Effusion."

ST. GEORGE'S HOSPITAL HUNTERIAN SOCIETY.—January 15th, Mr. Dent "History of Nursing at St. George's Hospital."

GUY'S HOSPITAL PUPILS' PHYSICAL SOCIETY.—January 10th, Clinical Evening. January 24th a paper by A. M. Daldy.

ST. MARY'S HOSPITAL MEDICAL SOCIETY.—January 7th, Medical Ethics, by Mr. W. E. Roughton. Clinical cases by Mr. J. C. Wood. January 21st, Antipyretics, by Mr. H. A. Caley. Clinical cases by Mr. G. A. Simmons.

MIDDLESEX HOSPITAL MEDICAL SOCIETY.—January 15th, Histological subject, Skin and Respiratory System. January 29th, Blood Vessels, Lymphatics, Ductless Glands.

ST THOMAS'S HOSPITAL MEDICAL AND PHYSICAL SOCIETY.—January 15th, Dr. Cullingworth on Oliver Wendell Holmes. January 29th, Mr. T. G. Nicholson, Mediæval Medicine.

UNIVERSITY COLLEGE MEDICAL SOCIETY.—January 14th, paper by Raymond Johnson, M.B., B.S., F.R.C.S. on the use of the Microscope in Surgery. Demonstrations—Pathological, Benign Tumours. Histological, Spermatogensis. January 28th, Short Papers. Demonstrations—Pathological Urine. Histological, Digestion and Deposition of Fat.

WESTMINSTER HOSPITAL GUTHRIE SOCIETY.—January 8th.

### Athletics.

FOOTBALL MATCHES.

ST. BARTHOLOMEW'S HOSPITAL.—Rugby, first fifteen, January 10th, v Middlesex Wanderers, at Richmond; 17th, v. Clapham Rovers, at Wandsworth: 21st, v. East Sheen, at Richmond; 24th, v. Old Leysians, Stamford Bridge; 31st, v. Old Merchant Taylors, at Balham; second fifteen, January 10th, v. Old Merchant Taylors, at Willesden Green; 17th, v. Eltham House, at Rayne's Park; 24th, v. Clapham Rovers, at Balham; 28th, v. St. Thomas's Hospital, at Balham; 31st, v. London Hospital, at Blackheath.

GUY'S HOSPITAL.—Rugby, first fifteen, January 3rd, England v. Wales, in Wales; 10th, v. Croydon, at Croydon; 17th, v. Old Leysians, at Stamford Bridge; 24th, v. Newport, at Newport; 31st, v. Rosslyn Park, at Raynes Park; second fifteen, January 10th, v. Croydon, at Raynes Park; 17th, v. Rosslyn Park, at Acton; 24th, Wickham Park, at Lee; 31st, v. Moore Park Wanderers, at Wood Vale; Association, January 10th, v. Vampires, at opponents' ground; 14th, v. Clapham Rovers, at Wandsworth; 17th, v. Caterham, at Raynes Park; 21st, v. Casuals, at Wormwood Scrubs; 24th, v. Barnes, at Raynes Park; 31st, v. Old Westminsters, at opponents' ground.

KING'S COLLEGE.—Association, January 17th, v. Queen's Park Rangers, at Kensal Green; 21st, 24th, 28th, v. Westminster Hospital, at Raynes Park; 31st, v. Polytechnic Wanderers, at Wimbledon; Rugby, January 24th, v. Ravens, at Regent's Park; 31st, v. Crusaders, at Ealing.

LONDON HOSPITAL.—Rugby, first fifteen, January 10th, v. Thurlow Park, at Streatham; 24th, v. Upper Clapton, at Clapton; 31st, v. Saracens, at Walthamstow; second fifteen, 10th, v. Thurlow Park, at home; 17th, v. Dental Hospital, away; 24th, v. Wasps, at home; 31st, v. St. Bartholomew's Hospital, II, at home. Association, January 10th, v. Old Salways, at Leytonstone; 17th, 24th, v. Barking Rovers, at Barking; 31st, v. West Croydon, at West Croydon.

ST. MARY'S HOSPITAL.—Rugby, first fifteen, January, 10th, v. Thornton Heath, at Thornton Heath; 17th, v. Wasps, at Putney; 21st, v. Old Epsomians, at home; 24th, v. Caledonians, at home; 28th, v. King's College, at home; 31st, v. Thurlow Park, at home. Second fifteen, January 10th, Thornton Heath, II, at home; 17th, v. Wasps, at home; 31st, v. Thurlow Park, II, at Streatham. Association, January 3rd, v. Condors, at Wimbledon; 14th, Old Westminsters, at Wimbledon; 17th, v. Polytechnic Wanderers, at Wimbledon; 31st, v. Civil Engineers, at Chiswick.

MIDDLESEX HOSPITAL.—First fifteen, January 3rd, 10th, 17th, v. Civil Service, at Richmond; 21st, v. London Welsh, at Balham; 28th, v. Lennox, at Dulwich; 31st.

ST. THOMAS'S HOSPITAL.—Rugby, first fifteen, January 10th, v. Kensington, at Wood Lane; 14th, v. East Sheen, at Richmond; 17th, v. Marlborough Nomads, at Surbiton; 24th v. Clapham, at Wandsworth; 31st v. Civil Service, at Richmond; second Fifteen, January 3rd; v. Rosslyn Park, 2nd fifteen, at Lambeth; 10th, v. Sidcup, at Sidcup; 17th v. Middlesex Wanderers 2nd fifteen, at Lambeth; 24th v. Blackheath 2nd fifteen, at Lambeth; 28th v. Barts 2nd fifteen, at Balham; 31st v. Harlequins 2nd fifteen, at Chiswick. Association, January 10th v. Bank of England, at home; 14th v. Old Wykehamists at home; 17th v. Reigate Priory, at Reigate; 24th v. Barnes, at home; 31st v. Surbiton Hill, at home.

UNIVERSITY COLLEGE AND HOSPITAL.—Association. First team, January 17th v. Royal School of Mines, at Barnes; 24th, v. Polytechnic Wanderers, at Wimbledon; Second team, January 17th, v. St. George's Hospital, at Neasden.

WESTMINSTER HOSPITAL.—Rugby, January 10th, v. Wandsworth, at Wandsworth; 17th, v. Clapham Park, at Eagle House; 24th, v. Christ's Hospital, at Raynes Park; 31st, v. Tooting College, at Raynes Park.

